# Research on the High Light Out-Coupling Efficiency Deep-Blue Top-Emitting Organic Light-Emitting Diode through FDTD Optical Simulation

**DOI:** 10.3390/nano13071282

**Published:** 2023-04-05

**Authors:** Saihu Pan, Suhao Hu, Bin Wei

**Affiliations:** 1 School of Microelectronics and Control Engineering, Changzhou University, Changzhou 213164, China; 2 Key Laboratory of Advanced Display and System Applications, Ministry of Education, Shanghai University, Shanghai 200072, China

**Keywords:** organic light-emitting diodes, FDTD, top-emitting, deep-blue, light out-coupling efficiency

## Abstract

We have studied high light out-coupling efficiency top-emitting organic light-emitting diodes (TOLEDs) under the guidance of the finite-difference time-domain (FDTD) simulation. TOLED achieves an extraordinarily high light extraction efficiency at 468 nm, in deep-blue regions, of 49.70%, which is approximately 3.5 times that of the bottom light-emitting diode (BOLED) by changing the thickness of the organic layer and the position of the light-emitting layer in the FDTD simulation. Based on the simulation results, the TOLED with ultrahigh efficiency and narrow full width at half maximum is successfully fabricated, and the maximum external quantum efficiency of TOLED is almost 3.3 times that of the BOLED, which is perfectly consistent with the FDTD simulation results. Meanwhile, the shift of the electroluminescence spectrum of the TOLED is restricted within 10° in the angular-dependence test (0° to 80°). The optimized performance of the OLED indicates a new method to develop a high-performance device under the guidance of simulation.

## 1. Introduction

Organic light-emitting diodes (OLEDs) have attracted much attention in recent years owing to their unique advantages of being lightweight, flexible, and environmentally friendly, and they have been utilized in the field of full-color displays and indoor lighting [1,2,3,4]. In order to realize the large-scale application of OLEDs in full-color monitors, obtaining high-efficiency red, green, and blue OLED devices is crucial. In particular, the deep-blue OLED device is the most worth studying because the efficiency of the deep-blue device is relatively low compared with the other two components. Improving the efficiency of a deep-blue light OLED device could significantly reduce the power consumption and extend the lifetime of a full-color monitor [5,6,7,8,9]. In recent years, many researchers have focused on achieving high-performance deep-blue OLEDs, mainly starting from the following two directions [10,11,12]. The first approach is synthesizing new materials, such as triplet–triplet annihilation (TTA) materials, heavy metal phosphorescence materials, and thermally activated delayed fluorescence materials, to enhance the internal quantum efficiency of devices [13,14,15,16]. The second route is designing new structures, such as semiconductor quantum wells, ultra-thin structures, and microcavity structures, to enhance the carrier balance or increase the light out-coupling efficiency [17].

On the other hand, because of the unique advantages of fast response, high contrast, and ultra-thinness, active-matrix organic light-emitting diodes (AMOLEDs) have become the first candidate in the field of high-quality image screens and flexible displays. In the application of AMOLED, the open ratio of top-emitting organic light-emitting diodes (TOLEDs) is significantly greater than that of bottom-emitting organic light-emitting diodes (BOLEDs) [18,19,20]. Furthermore, a typical TOLED device possesses a classical sandwich structure, and between the reflective bottom anode and the semitransparent upper cathode are organic layers. This enables quite a strong microcavity effect, bringing high light out-coupling efficiency by simply varying the organic layer’s thickness [21,22,23]. Therefore, in recent years, much research has been devoted to realizing high-performance TOLEDs. Most of the research focused on the overall structural optimization of devices and modifying the interfaces between different layers [24]. However, the traditional method is not suitable to design relatively complex structures, whereas more and more studies have turned to experiments accompanied by simulation. The finite-difference time-domain (FDTD) is an excellent method for handling Maxwell equations and is commonly used in numerical analysis for modeling in the field of electromagnetic fields, providing researchers with a unique perspective in solving electromagnetic and optical problems. Moreover, it is one of the most effective methods for solving complex optical issues in any type of structure [25,26]. With the help of simulation, the optical properties of OLEDs can be analyzed and designed more efficiently, and the results can be shown more quickly and easily than experiments, saving significant time and material costs.

In 2009, Qiang Wang et al. set the organic layer thickness of a green TOLED to 116 nm so that the device was located at the first-order microcavity length and obtained a maximum current efficiency (CE_max_) of 23.28 cd·A^−1^ and a maximum power efficiency (PE_max_) of 21.55 lm·W^−1^, approximately doubling the properties of the BOLED device [27]. In 2010, Simone Hofmann et al. set the red TOLEDs located at the first-, second-, and third-order microcavity length using FDTD to simulate the number of out-coupling photos. They also prepared a set of devices to test the angular dependence of their electroluminescence (EL) spectrum and the maximum external quantum efficiency (EQE_max_). Benefiting from excellent optical simulations, they achieved maximum EQEs of 29%, 17%, and 12%, respectively. However, the peak wavelength of the EL spectrum shifted obviously at different viewing angles, resulting in a significant deviation from commercial requirements [24]. In 2022, Yunping Zhao fixed the total thickness of the organic layer at 133 nm, allowing the red TOLED to be located at the first-order microcavity length with a CE_max_ of 28.27 cd·A^−1^, which was in great agreement with the light output intensity simulated by the FDTD method [28]. The spectrum has almost no transverse shift in different visual angles, which basically meets the commercial requirements. Although the efficiency of most of the red and green TOLEDs has already met commercial requirements after the optimization of the microcavity structure, the efficiency of blue devices, especially deep-blue devices, is still relatively low. In addition, most studies have focused on optimizing device efficiency by adjusting the microcavity length of the device, while little has been reported on the impact of the location of the light-emitting layer (EML) and the thickness of the top capping layer (CPL) on device efficiency for a fixed microcavity length.

In this work, we designed a structure of a TOLED device with a high light out-coupling efficiency of 49.70% by adjusting the thickness of the organic layer and the location of the EML. We applied a new deep-blue TTA emitter material 4,9-diisopropyl-N,N,N’,N’-tetrakis-(4-methyl-biphenyl-3-yl)-pyrene-1,6-diamine (DITBDAP) reported by our group previously in this structure [29]. The optical and electrical properties, such as EL spectrum, light out-coupling efficiency, and angular dependence characteristics were optimized under the guidance of FDTD, indicating that FDTD is an applicable tool for developing high-performance TOLED. Finally, the TOLED with high-efficiency, stable EL performance under different angles and narrow full width at half maximum (FWHM) have been realized by optimizing the optical structure. The optimized TOLED obtains an excellent EQE_max_ of 20.60% at the luminance of 1000 cd·m^−2^, which is 3.3 times as large as that of the BOLED. Meanwhile, the FWHM of the TOLED is significantly narrowed to 16 nm due to the microcavity effect.

## 2. Materials and Methods

**Figure 1:**
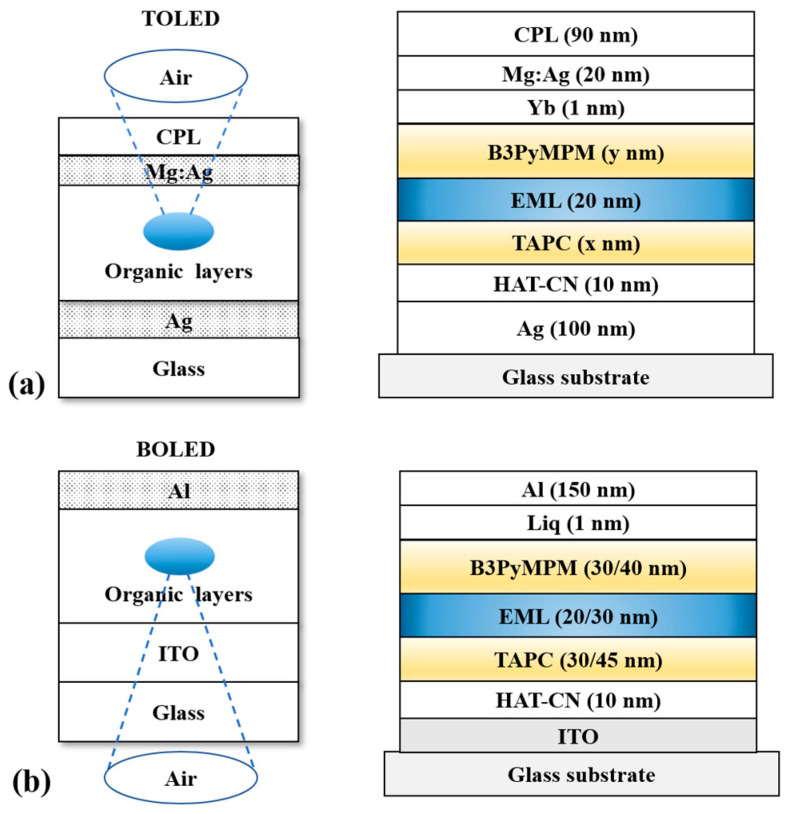
(**a**) Schematic diagram of TOLED; (**b**) schematic diagram of BOLED. The blue layer is the EML and the yellow layers are the charge transporting layers.

The software Lumerical FDTD Solutions 2018a is able to simulate micro- and nano-optical structures and non-linear optics in OLEDs accurately. The schematic diagrams of BOLED and TOLED are depicted in Figure 1. In the simulation, the refractive indices of glass and organic materials were supposed to be 1.51 and 1.75 at the wavelength of interest [27,30]. Al (150 nm) is used as the cathode and (quinolin-8-olato)lithium (Liq, 1 nm) is the cathode modification layer to reduce the potential barrier between the electron transport layer (ETL) and the cathode, facilitating the electron injection. The specific equations for the FDTD software calculations are shown in Formula S1 (Supplementary Information).

For the purpose of increasing the light out-coupling efficiency and the EQE of the TOLED, we varied the length of the microcavity consisting of organic layers, the position of the EML, and the thickness of the CPL in the FDTD simulation. The structure of the TOLED was as follows (Figure 1a): glass/Ag (100 nm)/1,4,5,8,9,11-Hexaazatriphenylenehexacarbonitrile (HAT-CN, 10 nm)/4,4’-cyclohexylidenebis[N,N-bis(4-methylphenyl)aniline] (TAPC, *x* nm)/EML (20 nm)/4,6-bis(3,5-di(pyridin-3-yl)phenyl)-2-methylpyrimidine (B3PyMPM, *y* nm)/Yb (1 nm)/Mg:Ag (9:1, 20 nm)/CPL (90 nm). TAPC is the hole transport layer (HTL) and B3PyMPM is the ETL. We varied the thicknesses of HTL and ETL, and the position of the EML to regulate the length of the microcavity. To compare and verify the high light out-coupling efficiency of TOLED, we designed BOLEDs with different thicknesses of organic layers as follows (Figure 1b): ITO/HAT-CN (10 nm)/TAPC (30/45 nm)/EML (20/30 nm)/B3PyMPM (30/40 nm)/Liq (1 nm)/Al (150 nm). FDTD was used to model and simulate the above device structures. The simulation steps include creating the physical model, setting the simulation area and geometry parameters, setting the excitation area and the monitor, and computing simulation and data analysis. Each layer was set as a rectangle of the same length (500 nm) and the widths were the same as those of the thicknesses of each layer in the device. In order to measure the total power generated in the EML, monitors were placed around the dipole source, and the perfectly matched layer (PML) boundary conditions were set on all boundaries to reduce the effect of light reflection. The angular dependence of the device was observed by calculating the far-field distribution of the emitting dipole source, and the near-field distribution was calculated to determine whether the light out-coupling efficiency was improved. EML was synthesized and all the other organic materials were purchased form Lumtech Corp. (Beijing, China) without any further purification.

## 3. Results and Discussions

### 3.1. FDTD Simulation of TOLEDs with Different Microcavity Lengths

We first calculated the optimized thickness of the organic layers. From an optical perspective, a TOLED could be simplified as a Fabry–Pérot optical resonance cavity with an electrically pumped emitting layer. The beam of light produced by the EML will be reflected back and forth indefinitely between the front mirror (cathode) and the rear mirror (anode), during which most of the light will be emitted and some will be dissipated. The microcavity effect will occur when the distance between the two electrodes is of the same order of magnitude as that of the wavelength of the light emitted by the EML. Only the light of a specific wavelength will be reinforced and transmitted through the semitransparent mirror (cathode). The intensity of the external luminescence spectrum (*I(λ)*) of a TOLED with a microcavity effect is shown below [31]:

(1) $$I(\lambda )=\frac{(1-R_{f})\left[1+R_{r}+2\sqrt{R_{r}}\cos(\frac{4\pi Z}{\lambda })\right]}{1+R_{f}R_{r}-2\sqrt{R_{f}R_{r}}\cos(\frac{4\pi L}{\lambda })}I_{0}(\lambda )$$

where *I*_0_*(λ)* is the intensity of the luminescence of the EML; *L* is the total optical thickness of the cavity (the sum of the thicknesses of organic layers); *λ* is the resonant wavelength; *Z* is the optical distance between the EML and anode; and *R*_f_ and *R*_r_ are the reflectivity of the cathode and the anode. For our TOLED, the light transmits through the front mirror (the cathode). In the case of metal as a reflector, the total optical thickness *L* of the cavity, taking into account the effective depth of penetration in the metal, is given by [32]:

(2) $$L=\sum n_{m}d_{m}+\left|\frac{\lambda }{4\pi }\sum_{i}^{}\Phi \right|$$

where *n*_m_ and *d*_m_ are the refractive index and thickness of organic material of the m^th^ layer in the microcavity. It is related to the reflected phase shift of the metal (*Φ*), and the phase shift of a metal mirror is expressed by the following equation [33]:

(3) $$\Phi =\arctan(\frac{2n_{s}b_{m}}{n_{s}{}^{2}-a_{m}{}^{2}-b_{m}{}^{2}})$$

where *a*_m_ and *b*_m_ are real and imaginary parts of the metal mirror refractive index and *n*_s_ is the refractive index of the material adjacent to the mirror. In order to maximize the *I(λ)*, the *L* of the microcavity device is also subject to the following equation:

(4) $$L=q\cdot \frac{\lambda_{q}}{2}$$

which is known as the Fabry–Pérot resonance equation, where *q* is the mode index and *λ*_q_ is the resonant wavelength of the series *q*. Since the total optical length is less than one optical wavelength, the order of the resonant cavity should be set to the lowest order. In the calculation, the refractive indices of organic materials were set as 1.75 and the complex refractive indices of the metal mirror were taken from [33]. Theoretical calculations based on the above equations have shown that the *I(λ)* could be maximized at the classical deep-blue light wavelength (about 464 nm) when the sum of the thicknesses of the organic layer is approximately 90 nm.

In order to verify this result, we used FDTD to simulate the emission intensity and light out-coupling efficiency of the TOLED at visible wavelengths. Meanwhile, it was assumed that the combined exciton in the EML could be represented as an oscillating dipole uniformly distributed in any direction, and that small variations in the decay rate of the excited state radiation were neglected. We varied the thickness of the HTL, so that the sums of the thicknesses of organic layers were 70 (device S1), 90 (device S2), and 110 nm (device S3), respectively, and the specific device structures are shown in Table 1.

**Figure 2:**
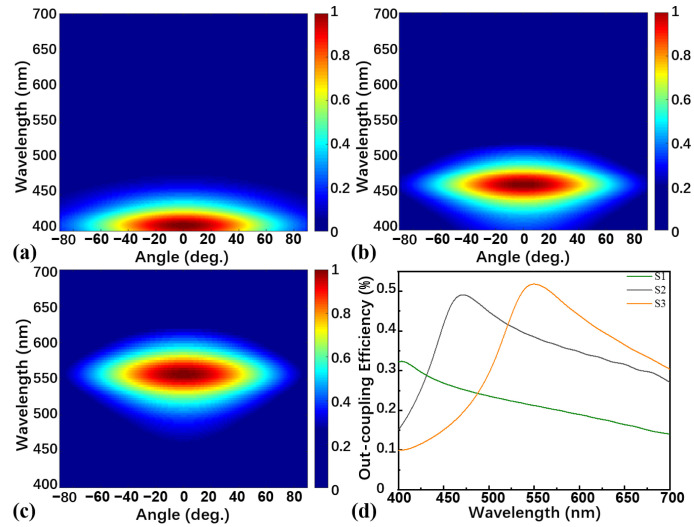
FDTD simulation of angle-wavelength-dependent normalized emission intensities of (**a**) device S1, (**b**) device S2, and (**c**) device S3; (**d**) FDTD simulation of light out-coupling efficiency of devices S1, S2, and S3.

Figure 2 displays the normalized emission intensities and light out-coupling efficiency at visible wavelengths for devices S1, S2, and S3, in which the peak of the spectral radiance energy of device S1 is at 404 nm (Figure 2a) and gradually redshifts to 549 nm as the thickness of the organic layers increases to 110 nm (device S3, Figure 2c). As can be seen from Figure 2b,d, the peak of the spectral radiance energy of device S2 is 471 nm, which is in line with theoretical calculations and is in the deep-blue light region. This phenomenon proves that our theoretical calculations are correct.

### 3.2. FDTD Simulation of TOLEDs with Different EML Positions and CPL Thickness

Given that the sum of the thicknesses of organic layers is determined, the position of EML will affect the efficiency of the device. The efficiency improvement of a TOLED could be expressed in terms of the radiation enhancement factor of the device at the resonant wavelength *λ*_q_, as follows [34]:

(5) $$G_{\mathrm{cav}}=\frac{\xi }{2}\cdot \frac{\left(1+R_{r}{)}^{2}(1-R_{f}\right)}{(1-\sqrt{R_{f}R_{r}}{)}^{2}}\cdot \frac{\tau_{\mathrm{cav}}}{\tau }$$

where *ξ* represents the enhancement factor related to the position of the EML, with a maximum value of 2 when the EML is just in the wave ventral region of the first-order microcavity. *τ*_cav_/*τ* is the ratio of the exciton lifetime in free space and in the microcavity device, it could be considered as a constant in the simulation. Therefore, we design devices S4 and S5 by adjusting the thickness of both ETL and HTL on the basis of the total thickness of organic layers fixed at 90 nm, the specific structures are shown in Table 1.

**Figure 3:**
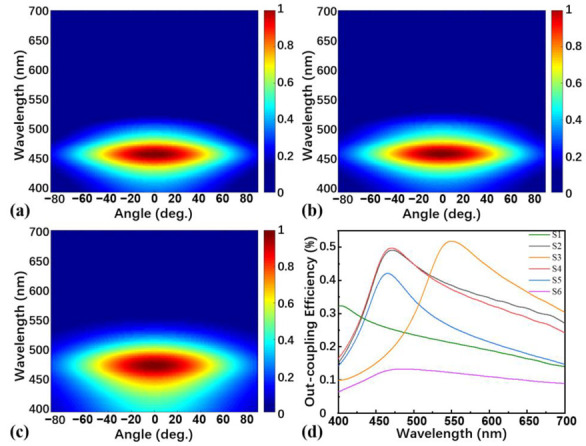
FDTD simulation of angle-wavelength-dependent normalized emission intensities of the device (**a**) S4, (**b**) S5, and (**c**) S6; (**d**) FDTD simulation of light out-coupling efficiency of all TOLED devices.

The spectral radiance energy of devices S4 and S5 are depicted in Figure 3a,b. It is shown that the shape and peaks differ slightly for devices S4 and S5, indicating that the position of EML (the distance between the EML and the Ag anode) does not affect the spectral radiance energy if the sum of the thickness of the organic layers is fixed at 90 nm. Furthermore, we obtained the out-coupling efficiency by calculating the ratio of the power transmitted into the air and to the total radiated power. Figure 3d concludes the out-coupling efficiencies of all TOLEDs. It can be seen that the out-coupling efficiency of device S5 is significantly lower than that of devices S2 and S4. The maximum out-coupling efficiencies of devices S2 and S4 are 49.08% at 471 nm and 49.70% at 468 nm, respectively. This indicates that the position of EML plays an important role in affecting the performance of the TOLED. Therefore, by determining the thickness of the sum of the thicknesses of organic layers and optimizing the position of the EML, we obtain device S4 with the highest out-coupling efficiency in the deep-blue light region.

In addition, to verify the effect of the CPL layer, we adjust the thickness of the CPL to 0 nm on the basis of device S4 and name it as device S6. The spectral radiance energy of device S6 shown in Figure 3c is significantly less concentrated compared to that of device S4 (Figure 3a) and the greatest out-coupling efficiency of device S6 is only 13.3%. This phenomenon is mainly due to the surface plasmon polariton effect at the interface between the metal and the air medium when the light in the EML propagates outwards, resulting in energy dissipation and a drop in light out-coupling efficiency, which could be suppressed by adding the CPL.

### 3.3. The Optical and Electronic Properties of TOLEDs and BOLEDs

**Figure 4:**
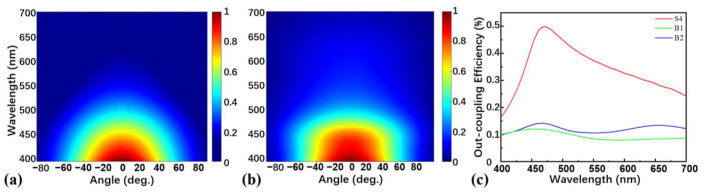
FDTD simulation of angle-wavelength-dependent normalized emission intensities of (**a**) device B1 and (**b**) device B2; (**c**) FDTD simulation of light out-coupling efficiencies of devices B1, B2, and S4.

In order to clarify whether the light out-coupling efficiency of TOLED device is improved compared to a BOLED, a BOLED named device B1 (Figure 1b) possessing the same layer structure as device S4 was designed, except that the 100 nm Ag anode was replaced by a 100 nm ITO, and the 20 nm Mg:Ag cathode was replaced by 150 nm Al, and the 1 nm Yb was replaced by 1 nm Liq as EIL. To achieve maximum light out-coupling efficiency and the best electrical performance, we have thickened the EML from 20 nm to 30 nm to obtain more excitons on the basis of device B1. We have also adjusted the HTL and ETL to 45 nm and 40 nm to facilitate hole and electron transmission and named the optimized device as device B2. The spectral radiance energy and the light out-coupling efficiency of devices B1 and B2 are simulated by FDTD, and the results are shown in Figure 4. As can be seen in Figure 4a,b, the spectral radiance energy of device B1 is similar to that of device B2. However, it is apparent from Figure 3a that the spectral radiance energy of device S4 is more concentrated than those of devices B1 and B2. In addition, Figure 4c compares the light out-coupling efficiency of devices B1, B2, and S4, in which the maximum efficiency of device B2 is 14.16%, slightly greater than that of device B1, while device S4 has a maximum efficiency of 49.70%, approximately 3.5 times larger than that of device B2.

**Figure 5:**
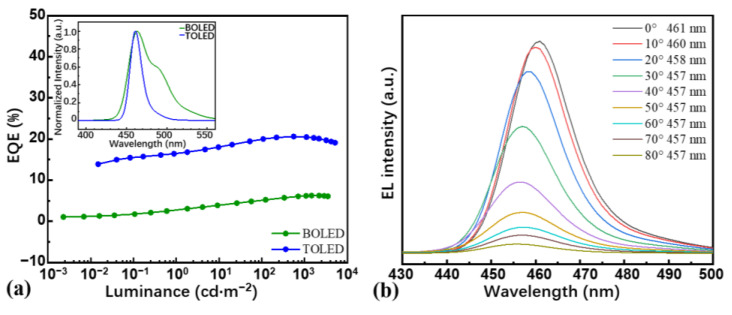
(**a**) EQE-luminance characteristics of the BOLED and TOLED and the EL spectrum as an inset; (**b**) the angular dependence test of the normalized light intensity with the EL emission spectra measured at different angles (0°–80°) [29].

Under the guidance of FDTD, BOLEDs and TOLEDs based on the new TTA material DITBDAP have been fabricated in the structure of devices S4 and B2, and the key parameters have been reported [29]. Both the BOLED and TOLED emitted deep-blue light with the EL emission peaks at 463 nm and 461 nm, respectively. The performance is summarized in Table 2. Figure S1 displays the *J*-*V* characteristics of BOLEDs and TOLEDs and Figure 5a describes the EQE and FWHM characteristics of BOLEDs and TOLEDs.

It is obvious in Figure 4b that the different emission structure has considerable effects on electro-photon conversion characteristics. The TOLED exhibits superior performance, with an EQE_max_ of 20.6%, about 3.3 times as high as the 6.1% obtained by BOLED, which further validates the FDTD simulation on the light out-coupling efficiency. The theoretical EQE calculation formula is as follows:

(6) $$\mathrm{EQE}=\gamma \times \Phi_{\mathrm{PL}}\times \eta_{\gamma }\times \eta_{\mathrm{op}}$$

where *γ* is the equilibrium constant of the carrier in the device, *Φ*_PL_ is the photoluminescence quantum yield (PLQY) of the molecules in the thin film state, *η*_γ_ is the radial exciton yield, ideally 100%, and *η*_op_ represents the light out-coupling efficiency of the device. Therefore, under the same structure of the organic layer, the high EQE_max_ of TOLED devices is mainly attributed to the excellent light out-coupling efficiency, consistent with the FDTD simulation results. As shown in the insert of Figure 5a, the emission peak wavelengths of the BOLED and TOLED are located at 463 nm and 461 nm, respectively, and the EL spectra exhibit almost identical characteristics, except for the difference in FWHM. The TOLED devices possess an extremely narrow FWHM of 16 nm, which is almost only a third of that of BOLED (43 nm). This phenomenon is mainly caused by the microcavity effect, which could reduce the FWHM of OLED devices obviously. The formula for calculating FWHM is Equation (7) [35], where FWHM has a direct inverse relationship with *L*. Within a certain range, as *L* increases, the FWHM will be significantly narrowed.

(7) $$\mathrm{FWHM}=\frac{\lambda^{2}}{2L}\cdot \frac{L-\sqrt{R_{f}R_{r}}}{\pi \sqrt[{4}]{R_{f}R_{r}}}$$

In order to test the angular dependence of the EL spectrum of TOLEDs, we have also measured the orientation pattern of the EL. It is clearly observed in Figure 5b that the peak wavelengths almost do not shift when the viewing angle increases from 0° to 80°, and the spectrum intensity of the device hardly changes within 10°, consistent with the angle-dependent spectral radiance energy results of the FDTD simulation. It indicates that this TOLED device is suitable for application in the field of direct-angle viewing displays, whose structure may be suitable for other deep-blue-emitting materials as well.

## 4. Conclusions

In summary, we have studied a light out-coupling efficiency deep-blue TOLED under the guidance of FDTD simulation. We optimized the performance of the TOLED by adjusting the thickness of the organic layer and the location of the EML and using the capping layer. In this design process, the organic layer thickness is one of the most crucial parameters. A thinner thickness of the organic layer leads to the blueshift of the spectral radiance energy and best light out-coupling efficiency, whereas a thicker one results in redshift. After determining the sum of the thicknesses of the organic layers to be 90 nm, we obtained a maximum light out-coupling efficiency of 49.70% by changing the position of the EML through FDTD, almost 3.5 times that of the BOLED. According to the simulation results of the FDTD, high-performance TOLEDs with EQE_max_ of 20.6% are successfully fabricated, indicating a new method to develop high-performance devices under the guidance of proper simulation.

## Figures and Tables

**Table 1:** FDTD simulation results of TOLEDs.

Device	Device Structure ^a^	*λ*_peak_ (nm) ^b^	Out-Coupling Efficiency (%)
S1	x = 20 nm, y = 20 nm, z = 90 nm	404	32.23
S2	x = 20 nm, y = 40 nm, z = 90 nm	471	49.08
S3	x = 20 nm, y = 60 nm, z = 90 nm	549	51.73
S4	x = 30 nm, y = 30 nm, z = 90 nm	468	49.70
S5	x = 40 nm, y = 20 nm, z = 90 nm	466	42.16
S6	x = 30 nm, y = 30 nm, z = 0 nm	510	13.30

^a^ x, y, and z present the thickness of HTL (TAPC), ETL, and CPL; ^b^ the wavelength at which the intensity of the luminescence is at the maximum.

**Table 2:** Summary of the performance of the BOLEDs and TOLEDs.

Device	*V*_on_ (V) ^a^	EQE_max_ (%)	FWHM (nm)	E_roll-off_ (%) ^b^
B2 (BOLED)	2.95	6.25	43	--
S4 (TOLED)	2.28	20.60	16	1%, 5%

^a^ Voltage corresponding to device luminance of 1 cd·m^−2^; ^b^ Efficiency roll-off at 1100 cd·m^−2^ and 3200 cd·m^−2^.

## Data Availability

The datasets used and analyzed in the current study are available from the corresponding author on reasonable request.

## Supplementary Materials

The following supporting information can be downloaded at: https://www.mdpi.com/article/10.3390/nano13071282/s1, Formula S1. The FDTD mechanism equations; Figure S1. The J-V curves of the BOLED and TOLED.
