# Supplementary material for: Research on the High Light Out-Coupling Efficiency Deep-Blue Top-Emitting Organic Light-Emitting Diode through FDTD Optical Simulation
Source: Nanomaterials (Basel). 2023 Apr 5;13(7):1282. doi: 10.3390/nano13071282 (PMC10097390; doi:10.3390/nano13071282)
Supplement: Supplementary file 1 [file nanomaterials-13-01282-s001.zip › nanomaterials-2287641-supplementary.pdf]

nanomaterials

Supplementary Information

Research on the High Light Out-Coupling Efficiency Deep-Blue Top-Emitting Organic Light-Emitting Diode through FDTD Optical Simulation

Saihu Pan [1,*], Suhao Hu [1] and Bin Wei [1,2,*]

[1] School of Microelectronics and Control Engineering, Changzhou University, Changzhou 213164, China; husuhao@shu.edu.cn
[2] Key Laboratory of Advanced Display and System Applications, Ministry of Education, Shanghai University, Shanghai 200072, China
* Correspondence: pansaihu@cczu.edu.cn (S.P.); bwei@shu.edu.cn (B.W.)

Formula S1. The FDTD Mechanism Equations.

In the scale of the free space, the difference form of Maxwell's equations is as follows:

$$\frac{\partial \vec{e}}{\partial t} = c\nabla \times \vec{h} \quad (1)$$

$$\frac{\partial \vec{h}}{\partial t} = -c\nabla \times \vec{e} \quad (2)$$

where c is the speed of light, $\vec{e}$ and $\vec{h}$ are the normalized results of electric and magnetic fields under free space impedance Z_0. Given that μ_0, and ε_0 are permeability and dielectric constant in free space, $\vec{e} = \vec{E} / Z_0$, $\vec{h} = \vec{H} / Z_0$, and $Z_0 = \sqrt{\mu_0 / \varepsilon_0}$. Equations (1-1) and (1-2) can also be written as six scalar equations in a three-dimensional planar rectangular coordinate system as follows:

$$\frac{\partial e_x}{\partial t} = c(\frac{\partial h_x}{\partial y} - \frac{\partial h_y}{\partial z})) \quad (3)$$

$$\frac{\partial e_y}{\partial t} = c(\frac{\partial h_x}{\partial z} - \frac{\partial h_z}{\partial x}) \quad (4)$$

$$\frac{\partial e_z}{\partial t} = c(\frac{\partial h_y}{\partial x} - \frac{\partial h_x}{\partial y}) \quad (5)$$

$$\frac{\partial h_x}{\partial t} = c(\frac{\partial e_y}{\partial z} - \frac{\partial e_z}{\partial y}) \quad (6)$$

$$\frac{\partial h_y}{\partial t} = c(\frac{\partial e_z}{\partial x} - \frac{\partial e_x}{\partial z}) \tag{7}$$

$$\frac{\partial h_z}{\partial t} = c(\frac{\partial e_x}{\partial y} - \frac{\partial e_y}{\partial x}) \tag{8}$$

According to the Yee's grid that discretizes the numerical value in free space, the definition of a point in space by Yee's grid is:

$$(i, j, k) = (i\Delta x, j\Delta y, k\Delta z) \tag{9}$$

where Δx, Δy, and Δz represent the varying distances of each grid cell in three coordinate directions. Equation $\Phi\big|_{i,j,k}^{n}$ for arbitrary time and space can be expressed as:

$$\Phi\big|_{i,j,k}^{n} = \Phi(x = i\Delta x, y = j\Delta y, z = k\Delta z, t = n\Delta t) \tag{10}$$

where Δt is the time step. Using the central difference approximating method with second order accuracy and discretizing the Yee's grid in time and space, the FDTD equations can be obtained as follows:

$$e_x\big|_{i+1/2,j,k}^{n+1} = e_x\big|_{i+1/2,j,k}^{n} + c\Delta t \cdot \left[\frac{h_z\big|_{i+1/2,j+1/2,k}^{n+1/2} - h_z\big|_{i+1/2,j-1/2,k}^{n+1/2}}{\Delta y} - \frac{h_y\big|_{i+1/2,j,k+1/2}^{n+1/2} - h_y\big|_{[illegible]}^{[illegible]}}{\Delta z} \right. \quad (111)$$

$$e_y\big|_{i,j+1/2,k}^{n+1} = e_y\big|_{i,j+1/2,k}^{n} + c\Delta t \cdot \left[\frac{h_x\big|_{i,j+1/2,k+1/2}^{n+1/2} - h_x\big|_{i,j+1/2,k-1/2}^{n+1/2}}{\Delta z} - \frac{h_z\big|_{i+1/2,j+1/2,k}^{n+1/2} - h_z\big|_{[illegible]}^{[illegible]}}{\Delta x} \right. \tag{12}$$

$$e_z\big|_{i,j,k+1/2}^{n+1} = e_z\big|_{i,j,k+1/2}^{n} + c\Delta t \cdot \left[\frac{h_y\big|_{i+1/2,j,k+1/2}^{n+1/2} - h_y\big|_{i-1/2,j,k+1/2}^{n+1/2}}{\Delta x} - \frac{h_x\big|_{i,j+1/2,k+1/2}^{n+1/2} - h_x\big|_{[illegible]}^{[illegible]}}{\Delta y} \right. \tag{13}$$

$$h_x\big|_{i,j+1/2,k+1/2}^{n+3/2} = h_x\big|_{i,j+1/2,k+1/2}^{n+1/2} + c\Delta t \cdot \left[\frac{e_y\big|_{i,j+1/2,k+1}^{n+1} - e_y\big|_{i,j+1/2,k}^{n+1}}{\Delta z} - \frac{e_y\big|_{i,j+1,k+1/2}^{n+1} - e_z\big|_{i,j,k+1[illegible]}^{n+1}}{\Delta y} \right. \tag{14}$$

$$h_y\big|_{i+1/2,j,k+1/2}^{n+3/2} = h_x\big|_{i+1/2,j,k+1/2}^{n+1/2} + c\Delta t \cdot \left[\frac{e_z\big|_{i+1,j,k+1/2}^{n+1} - e_y\big|_{i,j,k+1/2}^{n+1}}{\Delta x} - \frac{e_x\big|_{i+1/2,j,k+1}^{n+1} - e_x\big|_{i+1/2,[illegible]}^{n+1}}{\Delta z} \right. \tag{15}$$

$$h_z\big|_{i+1/2,j+1/2,k}^{n+3/2} = h_z\big|_{i+1/2,j+1/2,k}^{n+1/2} + c\Delta t \cdot \left[\frac{e_x\big|_{i+1/2,j+1,k}^{n+1} - e_x\big|_{i+1/2,j,k}^{n+1}}{\Delta y} - \frac{e_y\big|_{i+1,j+1/2,k}^{n+1} - e_y\big|_{i,j+1/[illegible]}^{n+1}}{\Delta x} \right. \tag{16}$$

These equations are preset in FDTD software and we used them to simulate our devices.

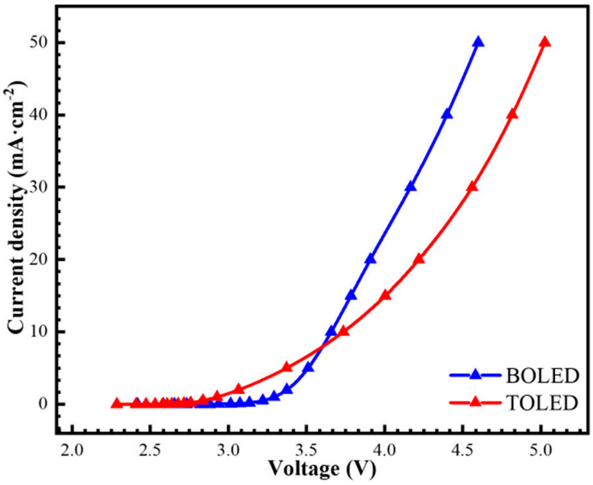

Figure S1. The J-V curves of the BOLED and TOLED.
